# Contribution of Inflammatory Cytokine Interleukin-18 Genotypes to Renal Cell Carcinoma

**DOI:** 10.3390/ijms20071563

**Published:** 2019-03-28

**Authors:** Wen-Shin Chang, Te-Chun Shen, Wei-Lan Yeh, Chien-Chih Yu, Hui-Yi Lin, Hsi-Chin Wu, Chia-Wen Tsai, Da-Tian Bau

**Affiliations:** 1 Terry Fox Cancer Research Laboratory, Translational Medicine Research Center, China Medical University Hospital, Taichung 40447, Taiwan; halittlemelon@hotmail.com (W.-S.C.); chestshen@gmail.com (T.-C.S.); wuhc@mail.cmuh.org.tw (H.-C.W.); 2 Institute of New Drug Development, China Medical University, Taichung 40402, Taiwan; wlyeh@mail.cmu.edu.tw; 3 School of Pharmacy, China Medical University, Taichung 40402, Taiwan; ccyu@mail.cmu.edu.tw (C.-C.Y.); hylin@mail.cmu.edu.tw (H.-Y.L.); 4 School of Medicine, China Medical University, Taichung 40402, Taiwan; 5 Graduate Institute of Biomedical Sciences, China Medical University, Taichung 40402, Taiwan; 6 Department of Bioinformatics and Medical Engineering, Asia University, Taichung 41354, Taiwan

**Keywords:** genotype, *IL-18*, polymorphism, renal cell carcinoma, Taiwan

## Abstract

Interleukin-18 (*IL-18*) is a multi-functional immuno-mediator in the development and progression of many types of infectious and inflammatory diseases. In this study, we evaluated the contribution of *IL-18* genotypes to renal cell carcinoma (RCC) in Taiwan via the genotyping of *IL-18* -656 (A/C), -607 (A/C), and -137 (G/C). Moreover, we analyzed their interactions with smoking, alcohol drinking, hypertension, and diabetes status. The results showed an association of the AC and CC genotypes of *IL-18* −607 with a significant decrease in the risk of RCC compared with the AA genotype (odds ratio (OR) = 0.44 and 0.35, 95% confidence interval (CI) = 0.27–0.72 and 0.18–0.66, *p* = 0.0008 and 0.0010, respectively). Furthermore, a significantly lower frequency of the C allele at -607 was observed in the RCC group (35.3% vs. 49.8%; OR = 0.53; 95% CI = 0.35–0.71, *p* = 0.0003). However, *IL-18* -656 and -137 did not exhibit a likewise differential distribution of these genotypes between the control and case groups. Stratifying the population according to smoking, alcohol drinking, hypertension, and diabetes status revealed a different distribution of *IL-18* -607 genotypes among non-smokers, non-drinkers, and patients without diabetes, but not among smokers, drinkers, or patients with diabetes. These findings suggest that *IL-18* -607 genotypes may play a role in the etiology and progression of RCC in Taiwan and may serve as a useful biomarker for early detection.

## 1. Introduction

Renal cell carcinoma (RCC) is the sixth most frequently diagnosed cancer in men (5%) and the tenth in women (3%) worldwide, thus posing a serious disease burden [1]. From an epidemiological viewpoint, RCC is the most common renal cancer and includes several subtypes that may be distinguished from each other by their histology, genetic background, clinical course, and response to treatment [2,3]. Moreover, there are several potential risk factors for RCC, including physical activity level, obesity, fruit and vegetable intake, cigarette smoking, and alcohol consumption. In addition, there are some common medical comorbidities for RCC, such as hypertension, diabetes, urinary stones, and other forms of chronic kidney diseases [3]. However, to date, no clinically practical genomic biomarker is available for RCC risk prediction. Unfortunately, many RCC patients, even those with advanced-stage tumors, remain asymptomatic [2,4], and the disease proceeds undetected. To make matters worse, up to 30% of RCC patients treated by radical nephrectomy suffer from many adverse effects and will relapse soon after their surgery [5]. Current personal prognostication of RCC is mainly based on histological validation, which may be labor intensive and time consuming, but frequently not useful for establishing a suitable course of treatment. Therefore, genomic molecular markers for early detection of RCC are urgently needed.

Interleukin-18 (IL-18), initially named IFN-γ inducing factor, is a proinflammatory cytokine encoded by the human *IL-18* gene and produced by activated macrophages, epithelial cells, osteoblasts, keratinocytes, and most importantly, cancer cells [6,7]. In syngeneic mice models, supplementary IL-18 administration suppressed the growth of Meth A sarcoma and mouse glioma cells [7,8,9], suggesting that IL-18 plays an essential role in host mechanisms of defense against tumors. On a molecular level, IL-18 homeostasis is under the control of highly complex machinery involved in chronic inflammation and carcinogenesis, which is of great interest to both immunologists and oncologists. However, the promotive or suppressive effects of IL-18 on carcinogenesis are not yet fully understood. First, IL-18 may exert its tumor-suppressive influences by stimulating IFN-γ production, promoting Th1 differentiation, enhancing the cytotoxic capacities of CD8+ lymphocytes and natural killer cells [7], inducing cancer cells to undergo programmed cell death [10], and suppressing the angiogenesis [7,11]. Second, IL-18 can inhibit the recognition of cancer cells by immune cells, enhancing cancer cell adherence to the vascular wall, increasing the production of angiogenic and growth factors, and providing a pro-metastatic microenvironment [12,13]. Third, the serum levels of IL-18 were found to be higher in cancer patients than in healthy subjects, including bladder cancer [14], ovarian cancer [15], gastrointestinal cancer [16], and non-small cell lung cancer [17]. Fourth, the serum levels of IL-18 were much higher in those from breast cancer patients with metastasis than in those from patients without metastasis and non-cancer healthy subjects, supporting the hypothesis that elevated serum IL-18 levels can be used as non-invasive markers for suspected metastatic potential [18]. All the above findings support the hypothesis that the pleiotropic cytokine IL-18 can biphasically exert both anti-cancerous and pro-cancerous activities [13]. To summarize, the molecular interactions of IL-18 and other molecules are very complex and deeply involved in tumorigenesis.

From a genomic perspective, the expression level of IL-18 appears to be determined by at least two single nucleotide polymorphisms (SNPs) in the promoter at positions -607 (A/C) and -137 (G/C) of the human *IL-18* gene. The former involves an A to C shift that disrupts a potential binding site for the cAMP responsive element binding protein, and the latter involves a G to C shift that abolishes the human histone H4 gene-specific transcription factor-1 (H4TF-1) nuclear factor binding site [19]. The alterations in transcription factor binding capacities determined by these two promoter polymorphisms may affect the overall activity of *IL-18*. Another SNP located in the *IL-18* promoter is -656 (A/C); however, the effects of different genotypes at *IL-18* -656 on cancer risk have not yet been well elucidated. That is to say, the contribution of -656 genotypes in human *IL-18* to autoimmune diseases has started to be examined [15], but their involvement in any type of cancer has not.

As mentioned above, most of the previous genomic studies on human *IL-18* were devoted to examining the association of *IL-18* -607 and -137 polymorphisms with various types of cancer. Some of them revealed positive associations with cancer risk [20,21,22,23,24], while others identified negative ones [25,26]. However, only one such study investigated the association of the -137 and -607 genotypes of *IL-18* with RCC [27]. In that paper, although the authors reported a negative association, they found that the -137 and -607 genotypes of *IL-18* were correlated with more advanced stages of RCC, and the genotype related to a higher production of IL-18 was associated with a larger size and T stage of the tumor [27]. In the present study, the promoter SNPs at positions -656 (A/C, rs1946519), -607 (A/C, rs1946518), and -137 (G/C, rs187238) of the *IL-18* gene were first examined and their genotype distributions analyzed in patients with RCC in Taiwan. In addition, we investigated the interaction of these *IL-18* promoter genotypes with personal behavioral and clinical factors that contribute to RCC susceptibility.

## 2. Results

### 2.1. Comparison of Characteristics Among Patients with Renal Cell Carcinoma (RCC) and Controls 

The frequency distributions in terms of age, gender, and personal behavioral habits for the 92 patients with RCC and the 580 cancer-free controls are summarized and compared in Table 1. Because the control subjects were already matched with patients with RCC for these factors, no difference was observed in terms of age and gender between these groups (*p* > 0.05). Moreover, there was no significant difference between the two groups in the frequency distributions in terms of personal behavioral habits, smoking, alcohol consumption, diabetes status, and family history (*p* > 0.05) (Table 1). An interesting finding was that there was a higher proportion of subjects with hypertension in the RCC group (66.3%) than in the cancer-free group (52.1%) (*p* = 0.0130). The percentage histologically identified as clear cell RCC patients is 77.2%. The percentages of “low” grade and “middle and high grade” are 52.2 and 47.8%, respectively (Table 1).

### 2.2. Analysis of the Association of IL-18 Promoter Genotypes and RCC Risk in Taiwan

The genotype frequencies of *IL-18* -656 (A/C, rs1946519), -607 (A/C, rs1946518), and -137 (G/C, rs187238) for the 92 patients with RCC and the 580 age- and gender-matched healthy control subjects were determined, and the comparative results of codominant, dominant, and recessive models are presented in Table 2. The frequencies of *IL-18* -656 and -137 genotypes in the control group, but not those of *IL-18* -656 (*p* = 0.0206), were in agreement with the Hardy–Weinberg equilibrium.

For the first time, the genotypes at the *IL-18* promoter -607 (A/C) polymorphic site were found to be differentially distributed between RCC cases and control groups (*p* for trend = 0.0004) (Table 2, middle panel). To explain in detail, the *IL-18* -607 heterozygous AC and homozygous CC genotypes were associated with decreased risks for RCC (OR = 0.44 and 0.35, 95% CI = 0.27–0.72 and 0.18–0.66, *p* = 0.0008 and 0.0010, respectively) (Table 2, middle panel). After adjusting for the potential confounders, including age, gender, smoking, alcohol consumption, hypertension, diabetes status, and family history status, the significances still existed (Table 2, middle panel). In the dominant and recessive analyzing models, a significant association with the risk for RCC still persisted, as observed for the homozygous CC genotype (Table 2, middle panel). In contrast, none of the genotypes or alleles for *IL-18,* -656, and -137 demonstrated any correlation with RCC risk in any of the subgroups (Table 2).

We further performed allelic frequency analysis for these three *IL-18* genotypes; the results are shown in Table 3. These results demonstrated that the variant allele C comprised only 35.3% in the RCC group, which was significantly less than that (49.8%) in the control group (adjusted OR = 0.53, 95% CI = 0.35–0.71, *p* = 0.0003), and these results fully confirmed the conclusion derived in Table 2. Consistently, the other two genotypes, *IL-18* -656 and -137, showed no significant association with the risk for RCC (Table 3).

### 2.3. Stratified Analysis of IL-18 Genotypes According to Personal Behavioral and Clinical Factors

**Figure 1:**
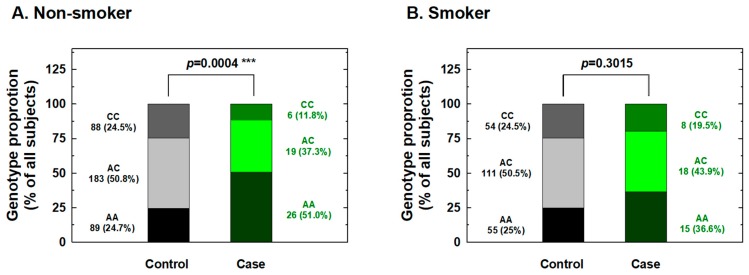
Contribution of *interleukin-18 (IL-18)* promoter -607 genotype to the risk of renal cell carcinoma after stratification by smoking status. The distributions of AA, AC, and CC genotypes at *IL-18* promoter -607 among nonsmokers (**A**) and smokers (**B**). *** Statistically significant between case and control groups.

**Figure 2:**
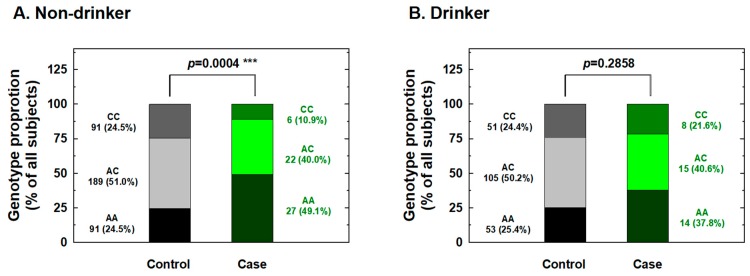
Contribution of *interleukin-18 (IL-18)* promoter -607 genotype to the risk of renal cell carcinoma after stratification by alcohol consumption status. The distributions of AA, AC, and CC genotypes at *IL-18* promoter -607 among nondrinkers (**A**) and drinkers (**B**). *** Statistically significant between case and control groups.

**Figure 3:**
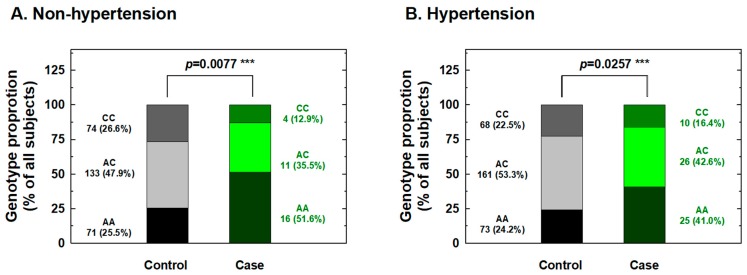
Contribution of *interleukin-18 (IL-18)* promoter -607 genotype to the risk of renal cell carcinoma after stratification by hypertension status. The distributions of AA, AC, and CC genotypes at *IL-18* promoter -607 among non-hypertensive (**A**) and hypertensive subjects (**B**). *** Statistically significant between case and control groups.

**Figure 4:**
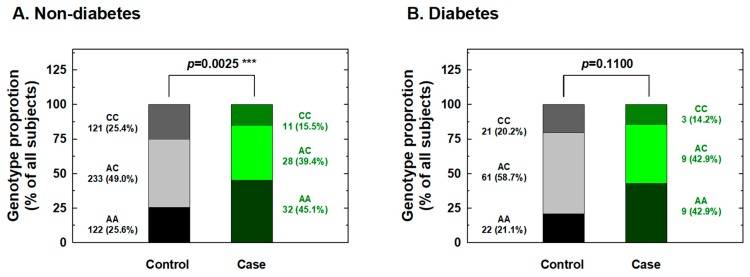
Contribution of *interleukin-18 (IL-18)* promoter -607 genotype to the risk of renal cell carcinoma after stratification by diabetes status. The distributions of AA, AC, and CC genotypes at *IL-18* promoter -607 among subjects without (**A**) and with (**B**) diabetes. *** Statistically significant between case and control groups.

We further conducted stratification analysis of the association between *IL-18* -607 genotypes and the risk for RCC based on potential personal behavioral and clinical risk factors among Taiwanese people, including cigarette smoking, alcohol consumption, hypertension, and diabetes status. First, the distributions of the genotype frequencies between the case and control groups among nonsmokers were significantly different, but showed similar proportions for cases and controls among smokers (Figure 1). The adjusted ORs for carriers with genotypes AC and CC at *IL-18* -607 were 0.36 and 0.22 for nonsmokers (95% CI = 0.21–0.63 and 0.11–0.56, respectively) and 0.61 and 0.58 for smokers (95% CI = 0.33–1.31 and 0.24–1.35, respectively), respectively (Figure 1). It appeared that the protective effects of *IL-18* -607 genotypes on the risk for RCC were obvious among nonsmokers, but not among smokers (Figure 1). Second, the distributions of the genotype frequencies between the case and control groups among nondrinkers were significantly different, but showed similar proportions for cases and controls among alcohol drinkers (Figure 2). The adjusted ORs for carriers with genotypes AC and CC at *IL-18* -607 were 0.38 and 0.21 among nondrinkers (95% CI = 0.22–0.70 and 0.09–0.51, respectively) and 0.56 and 0.62 among alcohol drinkers (95% CI = 0.31–1.23 and 0.28–1.41, respectively), respectively (Figure 2). The protective effects of *IL-18* -607 genotypes on the risk for RCC appeared to be obvious among nondrinkers, but not among alcohol drinkers (Figure 2). Third, the distributions of the genotype frequencies between case and control groups among non-hypertensive and hypertensive subjects were both significantly different (Figure 3). The adjusted ORs for carriers with genotypes AC and CC at *IL-18* -607 were 0.39 and 0.24 among subjects without hypertension (95% CI = 0.18–0.81 and 0.11–0.69, respectively) and 0.41 and 0.39 among those with hypertension (95% CI = 0.26–0.84 and 0.18–0.94, respectively), respectively (Figure 3). The protective effects of *IL-18* -607 genotypes on the risk for RCC appeared to be obvious among people with or without hypertension (Figure 3). Finally, the distributions of the genotype frequencies between the case and control groups among subjects without diabetes were significantly different, but presented similar proportions for cases and controls among subjects with diabetes (Figure 4). The adjusted ORs for carriers with genotypes AC and CC at *IL-18* -607 were 0.44 and 0.35 among subjects without diabetes (95% CI = 0.28–0.81 and 0.21–0.68, respectively) and 0.38 and 0.36 among those with diabetes (95% CI = 0.18–1.02 and 0.13–1.58, respectively), respectively (Figure 4). The effects of *IL-18* -607 genotypes on the risk for RCC appeared to be protective among subjects without diabetes, but not among those with diabetes (Figure 4). The sample size of those with a family history of cancer was too small for stratification analysis.

**Figure 5:**
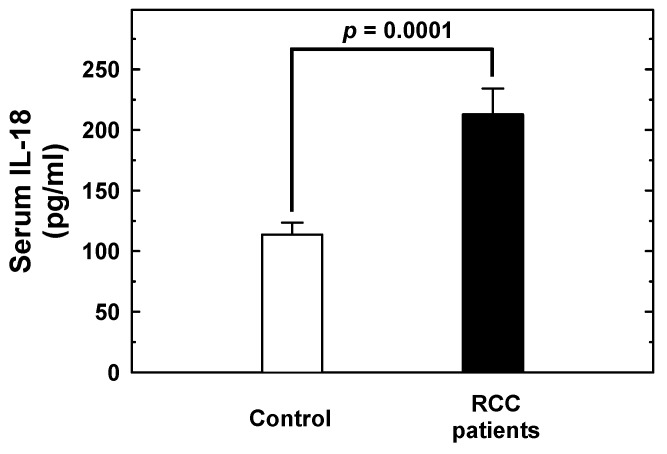
Serum IL-18 levels in 10 RCC patients and 10 healthy control subjects. The serum IL-18 levels were measured by ELISA methodology. The basal levels of serum IL-18 were higher in RCC patients (212.80 ± 21.39 pg/mL) than those of control subjects (113.70 ± 9.94 pg/mL) (*p* = 0.0001). However, no association was found between serum IL-18 levels and specific genotypes. The values are shown as mean ± standard deviation. IL-18, interleukin-18; RCC, renal cell carcinoma; ELISA, enzyme-linked immunosorbent assay.

The levels of IL-18 in the serum of 10 RCC patients and 10 healthy controls were determined using ELISA. The results demonstrated that the basal IL-18 levels were significantly higher in RCC patients (212.80 ± 21.39 pg/mL) than those of control subjects (113.70 ± 9.94 pg/mL) (*p* = 0.0001) (Figure 5). According to their *IL-18* -607 genotype distribution, the 10 RCC patients were divided into three subgroups: four patients with the AA genotype (216.25 ± 12.79 pg/mL), four with AC (206.25 ± 31.31 pg/mL), and two with CC (219.00 ± 19.80 pg/mL). There were no significant differences in serum IL-18 levels between different *IL-18* -607 genotypes (AC versus AA: *p* = 0.5759; CC versus AA: *p* = 0.8412; and CC versus AC: *p* = 0.6369).

## 3. Discussion

The prevalence and death rates of RCC are not ranked as high as those of other cancers in Taiwan. Clinically, surgery is the major course of RCC treatment. However, the symptoms of early-stage RCC are not obvious, and early detection of RCC is not available. Thus, the findings of genomic biomarker(s), which are very useful in rapid and convenient screening, may contribute to early detection and prediction of RCC susceptibility and outcome. For many years, members of the Terry Fox Cancer Fox Cancer Research foundation, including translational scientists and surgeons, have devoted themselves to elucidating specific and practical genomic biomarkers for early detection and prediction in Taiwan, where RCC is a prevalent condition and the cause of many cancer deaths [28,29,30,31,32]. Cytokines play an essential but complex role in the initiation and progression of inflammation and tumorigenesis [33], which is currently still under investigation. The proinflammatory cytokine IL-18 confers protective effects against cancer proliferation, such as that of lung cancer, in several murine models [13,34], and the benefits of recombinant human IL-18 have been shown in preclinical trials for cancer treatment [35]. However, despite the conventional view of IL-18 as an anticancer agent, some studies have also proposed a procancerous behavior for IL-18 under specific conditions [13]. Recently, mounting studies have reported that various cytokine genotypes may influence the serum levels of their counterpart cytokines, which may be closely associated with susceptibility to certain human diseases [19,20,21,22,23,25,26]. Among the numerous SNPs in *IL-18*, three polymorphisms are present in the promoter region of this gene: -656 (G/T), -607 (C/A), and -137 (G/C), and they were reported to cause differences in the transcription factor binding capacity and expression level of IL-18 in serum [19]. The polymorphic genotypes of *IL-18* promoter -607 and -137 were previously found to be associated with the risk of esophageal squamous cell carcinoma [20] and prostate cancer [21] in China, colorectal cancer in Greece [22], ovarian cancer in the USA (Hawaii) [23], and breast cancer in Iran [24]. On the contrary, there were also some negative associations reported between *IL-18* polymorphisms and the risk of head and neck cancers in Iran [26], as well as oral cancer in Greece [25]. Reasonable explanations for these discrepant and diverse findings may involve three possibilities: the dual impact of IL-18 on tumor-immune responses [13], the different types of cancer investigated, and variation among the populations under study [26].

In the current study, the genotypes at polymorphic locations -656 (G/T), -607 (C/A), and -137 (G/C) of the *IL-18* promoter region among RCC patients and healthy individuals in a Taiwan population were first determined and evaluated for their contribution to RCC risk. The results indicated a significantly lower risk for the heterozygous AC and homozygous CC variant genotypes and for the C allele at position -607 of the *IL-18* gene than their counterparts about RCC susceptibility (Table 2 and Table 3), even after statistical adjustment for personal behavioral and clinical risk factors. In contrast, no significant association between any genotype or allelic type with RCC risk was found for -656 or -137 of *IL-18* (Table 2 and Table 3). The positive findings indicating that genotypes of *IL-18* -607 may be determinants for personal RCC susceptibility were inconsistent with previous findings from the only paper investigating the contribution of *IL-18* genotypes to RCC [27], which returned negative findings. Once again, the inconsistency may be due to the location of Taiwan located in East Asia and the fact that it is an island with a conserved genetic, cultural, and environmental background, much different from the investigated Spanish population [27]. Although the sample size of the current study was similar to theirs (case:control = 92:580 vs. 158:506), we brought forward two novel findings: *IL-18* -607 was a determinant of RCC susceptibility (Table 2 and Table 3), and there were positive interactions of this polymorphic site with personal behavioral and clinical factors (Figure 1, Figure 2, Figure 3 and Figure 4). In the near future, the significant contribution of *IL-18* genotypes to RCC risk evaluation, especially those at *IL-18* -607, should be validated in larger samples and other populations worldwide.

In detail, the stratification of RCC patients and non-cancer subjects according to personal behavior revealed that *IL-18* -607 genotypes may play a significant role in the determination of susceptibility to RCC in non-smokers (Figure 1), non-alcohol drinkers (Figure 2), those with and without hypertension (Figure 3), and those without diabetes (Figure 4), but not in smokers, alcohol drinkers, or those with diabetes (Figure 1, Figure 3 and Figure 4). However, the undermined subtle mechanisms and signaling networks that are responsible for the interaction of IL-18 and other molecules related to the etiology of RCC require further investigation.

The genotype-phenotype association was performed after the measurement of serum levels of IL-18 in 10 RCC patients and 10 healthy controls. The results showed that: (a) the AA genotype at *IL-18* -607 was higher in the RCC patients than the healthy controls (Table 2); (b) the IL-18 levels were higher in the RCC patients than the healthy controls (Figure 5), which is consistent with the previous findings [36]; and (c) there was no difference in the IL-18 levels among RC patients of different *IL-18* -607 genotypes. This finding is consistent with the previous finding in lung cancer [37], but inconsistent with another [38]. The difference and similarity may due to the fact that different populations were investigated. Ours and the former were investigating Taiwanese and Chinese people, respectively, while the latter one was investigating people from Iran. 

As for the perspective molecular mechanism, there is literature mentioning that the A to C shift at IL-18 -607 may disrupt the potential binding site for the cAMP responsive element binding protein, thus lowering the expression level of IL-18 [19]. That is to say, the differential genotype at *IL-18* -607 may associate with elevated expression levels of IL-18, as the early detector for RCC, like we showed in Figure 5. The current study does not provide supporting evidence for the hypothesis that any genotype at *IL-18* -607 may associate with elevated expression levels of IL-18, due to the limited samples examined, and confirmation in larger samples is an urgent need. We also have to notice that the alteration of IL-18 may not be the only indicator during RCC carcinogenesis. In 2015, Xu et al. reported that elevated IL-18 together with IL-1b were significantly associated with advanced RCC stages, an elevated recurrence rate, and a shortened survival period among patients with localized RCC [35].

To summarize, this pilot study indicated a significant association between the *IL-18* -607 polymorphism and RCC in Taiwan. Furthermore, to the best of our knowledge, it is also the first to investigate the interaction of *IL-18* genotypes and behavioral and clinical factors in RCC risk. Our results showed a significant association between the *IL-18* -607 polymorphism and RCC, particularly in people without smoking or alcohol drinking behavior, and those without diabetes. However, it should be pointed out that the samples sizes of affected subjects (i.e., smokers, drinkers, and particularly patients with diabetes) were much smaller than those of un-affected subjects, which might be the main reason for the lack of statistically significant associations in the affected subjects. Future larger studies in various populations are needed to validate *IL-18* genotypes as early detective and predictive determinants of RCC.

## 4. Materials and Methods

### 4.1. Selected Subjects

This case-control study was performed in the China Medical University Hospital and involved the collection of data from 92 patients with RCC and 580 cancer-free controls matched by age and gender; none of the participants were related to each other by any biological relationship. The diagnosis of RCC, and the grades and types of each patient were histopathologically confirmed by the surgeons and pathologists led by Hsi-Chin Wu. In addition, the age- and gender-matched cancer-free controls were genetically unrelated to any of the recruited participants and had no prior history of any cancer. Originally, we frequency matched seven controls, which were collected in the Health Examination Center of the China Medical University Hospital, for each RCC patient with the same gender and age at ±2 years. After the first-term matching, those with incomplete demographic data about smoking, alcohol drinking status, hypertension, diabetes, or family cancer history, were excluded. A further exclusion criterion for the control subjects was any symptom suggestive of RCC, such as hematuria. Finally, only 580 controls were collected in the study. After obtaining written informed consent, 3–5 mL of venous blood was collected from each participant for genotyping. The study was approved by the Institutional Review Board of China Medical University, and expert members of the Tissue Bank of China Medical University Hospital (DMR98-IRB-209 in 2009) provided their kind assistance. The overall agreement rate among the participants was >85%. Select characteristics of all the participants are summarized and compared in Table 1.

### 4.2. DNA Preparation and Storage

Genomic DNA from the leukocytes of each study subject was extracted using the QIAamp Blood Mini Kit (Qiagen, Valencia, CA, USA), stored for the long term at −80 °C, simultaneously diluted, and aliquoted and stored for genotyping as a working stock at −20 °C, as we have frequently performed previously [32,39,40].

### 4.3. IL-18 Genotype Discrimination Methodology

The genotype discrimination methodology for *IL-18* -137, -607, and -656 genotypes was performed as we described in 2018 [41]. Briefly, -137 (G/C, rs187238) and -607 (A/C, rs1946518) genotyping was performed using the ABI StepOne™ Real-Time PCR System (Applied Biosystems, Foster City, CA, USA) and analyzed using the typical TaqMan assay. Regarding the genotyping of *IL-18* -656 (A/C, rs1946519), the polymerase chain reaction-restriction fragment length polymorphism (PCR-RFLP) methodology was carried out using the primers originally reported in 2005 by Flowaczny et al. [42], with the forward primer being 5′-AGGTCAGTCTTTGCTATCATTCCAGG-3′ and the reverse primer being 5′-CTGCAACAGAAAGTAAGCTTGCGGAGAGG-3′, and a 120-bp fragment nearby the *IL-18* -656 polymorphism was amplified. In detail, approximately 100 ng of genomic DNA of each sample was subjected to PCR, in which the reaction mixture of 25-μL contained 300 mM dNTP, 2 U of Taq DNA polymerase, 1× PCR buffer, 1.5 mM MgCl_2_, and 0.8 mM of each primer. After mixing up and briefly spinning down, the reaction mixture was heated to 94 °C for 4 min and amplified by 30 cycles using the My Cycler (Biorad, Hercules, CA, USA) with the following steps: denaturation at 94 °C for 60 s, annealing at 60 °C for 60 s, extension at 72 °C for 60 s for each cycle, and a final extension step at 72 °C for 5 min. The volume of the restriction assay was set at 12.5 μL, containing 8 μL of PCR products, 2 U *Mwo* I restriction enzyme, and 1× buffer. The reaction mixture was then incubated for 16 h or overnight at 60 °C. The resultant DNA fragments were subject to electrophoresis in 3.0% agarose gel at 100 V for 30 min. After electrophoresis, ethidium bromide staining was done to observe the DNA fragments under UV (260 nm) light. For the A allele of *IL-18* -656, there was no digestion of the 120-bp PCR fragment, whereas for the C allele of *IL-18* -656, two (96- and 24-bp) fragments were identified.

### 4.4. Enzyme-linked Immunosorbent Assay (ELISA) for Serum IL-18 Levels

Ten milliliters of blood samples were collected from 10 healthy controls and 10 RCC patients. The blood samples were collected in serum tubes with an accelerating agent for serum separation and kept at a room temperature for 30 min before their further centrifugation for 20 min at 1500× g. Serum was then isolated and stored at −80 °C until IL-18 measurement. The individual level of IL-18 in serum was measured by enzyme-linked immunosorbent assay (ELISA, Newwark, DE, USA) kits.

### 4.5. Statistical Analysis Methodology

The data of 580 cancer-free healthy controls and 92 patients with RCC who had complete genotypic and clinical details were finally included for statistical analysis, whose results are presented in the form of tables and figures. To ensure that the control subjects in this study were representative of the Taiwanese general population and to exclude the possibility of genotyping error, the deviation of the genotype frequencies of *IL-18* SNPs in the control subjects from those expected under the Hardy–Weinberg equilibrium was assessed using the goodness-of-fit test. Pearson’s Chi-square test was used to compare the distribution of *IL-18* genotypes between cases and control groups and in the stratification analysis. The comparison of the continuous factor age was performed and evaluated by the Student’s *t*-test. The contribution of *IL-18* genotypes to the risk of developing RCC was estimated by the odds ratios (ORs) and their counterpart 95% confidence intervals (CIs) obtained through logistic regression analysis with adjustment for possible confounders. Any *p* value < 0.05 was considered to be statistically significant.

## Figures and Tables

**Table 1:** Distributions of the frequencies of selected characteristics among the renal cell carcinoma (RCC) cases and healthy controls.

Characteristics	Cases (*n* = 92)			Controls (*n* = 580)		*p*-Value
	N	%		N	%	
Age (year) (mean ± SD)	58.8 ± 11.7			58.3 ± 11.5		0.8971
≤60	47	51.1%		307	52.9%	0.8223
>60	45	48.9%		273	47.1%	
Gender						
Male	59	64.1%		371	64.0%	1.0000
Female	33	35.9%		209	36.0%	
Smoking status						
Smokers	41	44.6%		220	37.9%	0.2499
Non-smokers	51	55.4%		360	62.1%	
Alcohol drinking status						
Drinkers	37	40.2%		209	36.0%	0.4848
Non-drinkers	55	59.8%		371	64.0%	
Hypertension						
Yes	61	66.3%		302	52.1%	0.0130 *
No	31	33.7%		278	47.9%	
Diabetes						
Yes	21	22.8%		104	17.9%	0.2523
No	71	77.2%		476	82.1%	
Family cancer history						
Yes	6	6.5%		17	2.9%	0.1125
No	86	93.5%		563	97.1%	
Histological types						
Clear cell	71	77.2%				
Non-clear cell	21	22.8%				
Histological grades						
Low	48	52.2%				
Middle and high	44	47.8%				

* Statistically identified as significant based on Chi-square test without Yates’ correction.

**Table 2:** Distribution of *interleukin-18 (IL-18)* genotypes among the renal cell carcinoma patients and non-cancer healthy control subjects.

Genotypes	Controls			Patients			OR (95% CI) ^a^			aOR (95% CI) ^a^	*p*-Value
	*n*	%		*n*	%						
***IL-18* -656**											
AA	221	38.1%		32	34.8%		1.00 (Reference)			1.00 (Reference)	
AC	252	43.5%		44	47.8%		1.21 (0.74–1.97)			1.20 (0.69–1.84)	0.4535
CC	107	18.4%		16	17.4%		1.03 (0.54–1.96)			1.04 (0.52–1.93)	0.9218
*P*_trend_											0.7311
Carrier comparison											
AA + AC	473	81.6%		76	82.6%		1.00 (Reference)			1.00 (Reference)	
CC	107	18.4%		16	17.4%		0.93 (0.52–1.66)			0.96 (0.56–1.46)	0.8076
AA	221	38.1%		32	34.8%		1.00 (Reference)			1.00 (Reference)	
AC + CC	359	61.9%		60	65.2%		1.15 (0.73–1.83)			1.12 (0.70–1.79)	0.5414
										
***IL-18* -607**											
AA	144	24.8%		41	44.6%		1.00 (Reference)			1.00 (Reference)	
AC	294	50.7%		37	40.2%		0.44 (0.27–0.72)			0.41 (0.22–0.64)	0.0008 *
CC	142	24.5%		14	15.2%		0.35 (0.18–0.66)			0.33 (0.15–0.58)	0.0010 *
*P*_trend_											0.0004 *
Carrier comparison											
AA + AC	438	75.5%		78	84.8%		1.00 (Reference)			1.00 (Reference)	
CC	142	24.5%		14	15.2%		0.55 (0.30-1.00)			0.51 (0.32–0.96)	0.0505
AA	144	24.8%		41	44.6%		1.00 (Reference)			1.00 (Reference)	
AC + CC	436	75.2%		51	55.4%		0.41 (0.26–0.65)			0.39 (0.25–0.66)	0.0001 *
										
***IL-18* -137**											
GG	463	79.8%		72	78.3%		1.00 (Reference)			1.00 (Reference)	
GC	108	18.6%		18	19.5%		1.07 (0.61-1.87)			1.06 (0.60-1.66)	0.8074
CC	9	1.6%		2	2.2%		1.42 (0.30-6.75)			1.37 (0.35-6.23)	0.6505
*P*_trend_											0.8825
Carrier comparison											
GG + GC	571	98.4%		90	97.8%		1.00 (Reference)			1.00 (Reference)	
CC	9	1.6%		2	2.2%		1.41 (0.30-6.63)			1.38 (0.32-5.98)	0.6622
GG	463	79.8%		72	78.3%		1.00 (Reference)			1.00 (Reference)	
GC + CC	117	20.2%		20	21.7%		1.10 (0.64-1.88)			1.10 (0.65-1.85)	0.7289

OR: odds ratio; ^a^ The ORs were estimated with multivariate logistic regression analysis after being adjusted with age, gender, smoking, alcohol drinking, hypertension, diabetes, and family history status; * Statistically identified as significant based on Chi-square test without Yates’ correction.

**Table 3:** Allelic frequency analysis for *interleukin-18 (IL-18)* polymorphisms and renal cell carcinoma.

Allele	Controls *n* (%)	Patients *n* (%)	aOR (95% CI) ^a^	*p*-Value
***IL-18* -656**				
G	694 (59.8%)	108 (58.7%)	1.00 (Reference)	
T	466 (40.2%)	76 (41.3%)	1.06 (0.73-1.31)	0.7712
***IL-18* -607**				
A	582 (50.2%)	119 (64.7%)	1.00 (Reference)	
C	578 (49.8%)	65 (35.3%)	0.53 (0.35-0.71)	0.0003 *
***IL-18* -137**				
G	1034 (89.1%)	162 (88.0%)	1.00 (Reference)	
C	126 (10.9%)	22 (12.0%)	1.11 (0.69-1.83)	0.6595

^a^ The ORs were estimated with multivariate logistic regression analysis after being adjusted with age, gender, smoking, alcohol drinking, hypertension, diabetes, and family history status. * Statistically identified as significant based on chi-square test without Yates’ correction.
